# Penicitroamide, an Antimicrobial Metabolite with High Carbonylization from the Endophytic Fungus *Penicillium* sp. (NO. 24)

**DOI:** 10.3390/molecules21111438

**Published:** 2016-10-28

**Authors:** Zi-Wei Feng, Meng-Meng Lv, Xue-Shuang Li, Liang Zhang, Cheng-Xiong Liu, Zhi-Yong Guo, Zhang-Shuang Deng, Kun Zou, Peter Proksch

**Affiliations:** 1 Hubei Key Laboratory of Natural Product Research and Development, College of Biological and Pharmaceutical Sciences, China Three Gorges University, Yichang 443002, China; fzw080907@gmail.com (Z.-W.F.); MengmengLv828@yeah.net (M.-M.L.); lixueshuang1205@163.com (X.-S.L.); zhangliang@hec.cn (L.Z.); liuchengxiong666@126.com (C.-X.L.); kzou@ctgu.edu.cn (K.Z.); 2 Institut für Pharmazeutische Biologie und Biotechnologie, Heirich-Heine University Dusseldorf, Universitätsstr. 1, Gebäude 26.23, Dusseldorf 40225, Germany; Peter.Proksch@uni-duesseldorf.de

**Keywords:** endophytic fungus, penicitroamide, *Penicillium* sp., antibacterial activity, brine shrimp lethality

## Abstract

Penicitroamide (**1**), a new metabolite with a new framework, was isolated from the ethyl acetate extract of the PDB (Potato Dextrose Broth) medium of *Penicillium* sp. (NO. 24). The endophytic fungus *Penicillium* sp. (NO. 24) was obtained from the healthy leaves of *Tapiscia sinensis* Oliv. The structure of penicitroamide (**1**) features a bicyclo[3.2.1]octane core unit with a high degree of carbonylization (four carbonyl groups and one enol group). The chemical structure of penicitroamide (**1**) was elucidated by analysis of 1D-, 2D-NMR and MS data. In bioassays, penicitroamide (**1**) displayed antibacterial potency against two plant pathogens, *Erwinia carotovora* subsp. Carotovora (Jones) Bersey, et al. and *Sclerotium rolfsii* Sacc. with MIC_50_ at 45 and 50 μg/mL. Compound **1** also showed 60% lethality against brine shrimp at 10 μg/mL. Penicitroamide (**1**) exhibited no significant activity against A549, Caski, HepG2 and MCF-7 cells with IC_50_ > 50 μg/mL. Finally, the possible biosynthetic pathway of penicitroamide (**1**) was discussed.

## 1. Introduction

Endophytic fungi from plants can yield an extremely large amount of natural products with unique frameworks including high biological potency and low side effects [1,2,3,4,5,6,7]. Natural products scientists focus most of their attention to the secondary metabolites derived from plant endophytic fungi rather than from the traditional medicinal plants or folk medicines. Meanwhile, genetic biologists are involved in the whole genome sequencing of the filamentous fungi genera—*Aspergillus*, *Penicillium* and *Fusarium* [8]. Currently we can find out which gene clusters control the biosynthesis of natural products using methods of biological information analysis. Thus, there is great potency for controllable fungi natural products manufacturing using genetic engineering technology. In our ongoing search for new bioactive secondary metabolites from plant endophytic fungi in the Shennongjia District [9,10,11,12], a chemical investigation of an endophytic *Penicillium* sp. (NO. 24), isolated from the healthy leaves of *Tapiscia sinensis* Oliv., was performed, and led to the isolation of a new alkaloid with a new bicyclo[3.2.1]octane core structural framework—penicitroamide (**1**). Interestingly, there are four carbonyl groups and a stable enol group in penicitroamide (**1**), which is rare in natural products. Its structure was proven by the analysis of 1D-, 2D-NMR, EI-MS, HR-EI-MS and CD data. Herein, we reported the isolation, structure elucidation, and biological activities of penicitroamide (**1**).

## 2. Results

### 2.1. Structural Elucidationof Penicitroamide *(**1**)*

Penicitroamide (**1**) was obtained as a brown oil, ${\left[\alpha \right]}_{D}^{25}$ = −19 (*c*, 0.01). Its molecular formula was determined as C_29_H_39_NO_6_ by HR-EI-MS with *m/z* 497.2775 (calcd. 497.2777). From the results of mass spectra, there was (2n + 1) nitrogen atoms in compound **1** according to the Nitrogen Rule of the mass spectrum. Simultaneously, 11 degrees of unsaturation were obtained from the molecular formula of compound **1**, and three carbonyl groups, one ester or amide carbonyl group and eight olefinic carbons in the carbon resonances were due to eight degrees of unsaturation; thus, the three remaining unsaturation degrees can be ascribed to the three aliphatic rings in penicitroamide (**1**).

There were five methyl groups, six methylene groups, 10 methyne groups and eight quaternary carbons, including three carbonyl groups and one ester or amide carbonyl group at δ 208.5 (C-7), l97.3 (C-11), 206.8 (C-20) and 170.7 (C-18) in the ^13^C- and DEPT-NMR spectra of penicitroamide (**1**). The five methyl groups were observed at 1.85 (d, 6.0)/18.6 (C-17), 1.59 (s)/17.7 (C-27), 0.99 (s)/10.4 (C-28), 1.07 (s)/23.4 (C-29), and 1.11 (d, 6.1)/13.3 (C-30) in the ^1^H-NMR spectrum of penicitroamide (**1**). There were six olefinic protons signals at 6.66 (d, 14.6) (H-13), 7.27 (dd, 11.0, 14.0) (H-14), 6.39 (d, 11.4) (H-15), 6.28 (m) (H-16), 5.36 (br s, 2H) (H-25, 26) and eight olefinic carbons at 167.8 (C-12), 142.3 (C-14), 139.7 (C-16), 131.1 (C-15, 25), 124.5 (C-26), 118.6 (C-13), 108.9 (C-10) in the 1D-NMR and HSQC spectra.

**Figure 1:**
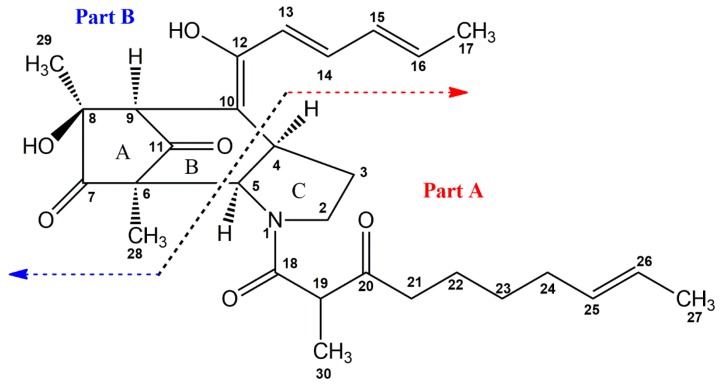
The structure of penicitroamide (**1**).

The structure of **1** was established by the comprehensive analysis of the 2D-NMR spectra including ^1^H-^1^H COSY, HSQC and HMBC. In the ^1^H-^1^H COSY spectrum, there appeared four substructures in compound **1**, −2.39 (H-21)/1.39 (H-22)/1.23 (H-23)/1.89 (H-24)/5.36 (H-25, 26)/1.59 (H-27), −6.66 (H-13)/7.27 (H-14)/6.39 (H-15)/6.28 (H-16)/1.85 (H-17), −3.71 (H-2)/2.10, 1.50 (H-3)/3.35 (H-4)/4.65 (H-5) and −3.83 (H-19)/1.11 (H-30), which were proven by the corresponding HMBC cross peaks. The following correlations from 1.11 (H-30) to 50.9 (C-19), 170.7 (C-18) and 206.8 (C-20), from 3.83 (H-19) to 170.7 (C-18) and 206.8 (C-20), from 2.39 (H-21) to 50.9 (C-19) and 206.8 (C-20) and from 1.39 (H-22) to 206.8 (C-20) elongated the substructure from C-21 to C-18. Both the cross peaks between 170.7 (C-18) and 4.65 (H-5), 2.75 (H-2b) in the HMBC spectrum and the chemical shifts of C-2 and C-5 at δ 47.6 and 61.2 hinted that there may be a nitrogen atom among the C-2, C-5 and C-18, which was verified by the HR-EI-MS. Additionally, from the correlations of 4.65 (H-5) to 47.6 (C-2), 29.2 (C-3) and 34.9 (C-4), from 3.71/2.75 (H-2) to 34.9 (C-4) and 61.2 (C-5), and from 1.50 (H-3) to 61.2 (C-5), the substructure of part A was inferred (see Figure 1 and Supplementary Materials). A similar structural subunit of part A was found in Perinadine A and Scalusamide A [13,14]. Comparing their ^1^H- and ^13^C-NMR data, the chemical shifts of C-2-C-5, C-18-C-27 and C-30 in penicitroamide (**1**) were similar to those in Perinadine A and Scalusamide A, considering the different chemical surroundings of C-4 and C-5 and the deuterium solvent effect between DMSO-*d*_6_ and CDCl_3_. The *E*-geometry of the double bond was hinted at by the References [13,14], because the two olefinic protons on C-25 and C-26 were overlapped in penicitroamide (**1**).

In the ^1^H-^1^H COSY spectrum, there were cross peaks from 6.66 (H-13) to 7.27 (H-14), from 7.27 (H-14) to 6.39 (H-15), from 6.39 (H-15) to 6.28 (H-16) and from 6.28 (H-16) to 1.85 (H-17), which revealed that there was a substructure of -C-13-C-14-C-15-C-16-C-17 in penicitroamide (**1**). Along the above substructure, the carbon chain was extended to C-12 and C-10, because of the correlations from 6.66 (H-13) to 167.8 (C-12) and 108.9 (C-10) and from 7.27 (H-14) to 167.8 (C-12). The cross peaks of 3.29 (H-9)/108.9 (C-10), 167.8 (C-12), 1.07 (H-29)/208.5 (C-7), 73.2 (C-8), 45.7 (C-9), 6.99 (H-28)/167.0 (C-6), 208.5 (C-7), 197.3 (C-11), 6.12 (8-OH)/208.5 (C-7), 73.2 (C-8), 45.7 (C-9), 23.4 (C-29) suggested that there was a five-membered ring including C-6, C-7, C-8, C-9 and C-11 and the relative locations of those five carbons in compound **1**. Lastly, the cross peak of 3.29 (H-9)/108.9 (C-10) proved that the five-membered ring was linked to the above prolonged side chain by C-9. Thus, the structure of part B from compound **1** was established as shown in Figure 1. The *E*-geometry of the double bonds was suggested by the large *J* (H-13/H-14, H-15/H-16) values at 14.6 and 11.0 Hz.

The completely planar structure of penicitroamide (**1**) was confirmed by the following correlations: from 4.65 (H-5) to 107.0 (C-6), 197.3 (C-11), 0.99 (H-28)/61.2 (C-5) and 3.35 (H-4)/45.3 (C-9) and 108.9 (C-10), in which part A and B formed a bridge-ring, not a fused-ring. The A ring and B ring comprised a bicyclo[3.2.1]octane core unit through C-6 and C-9, and the six-membered ring (B ring) was fused with the five-membered ring containing a nitrogen atom at C-4 and C-5. Finally, the planar structure of compound **1** was built, a new carbon skeleton named penicitroamide (**1**).

**Figure 2:**
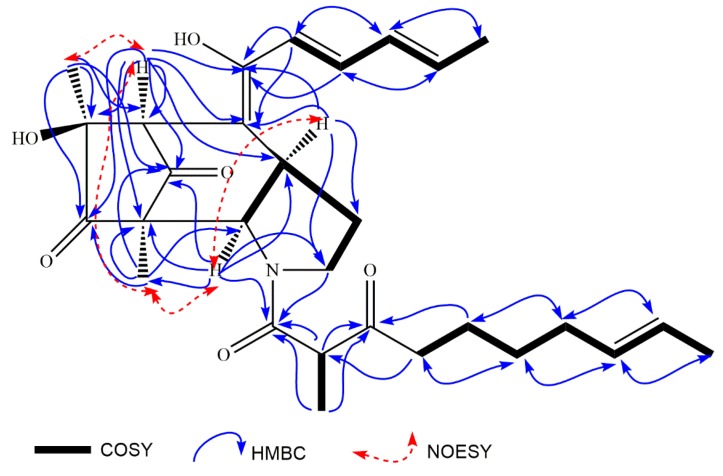
The key COSY, HMBC and NOESY correlations of penicitroamide (**1**).

**Figure 3:**
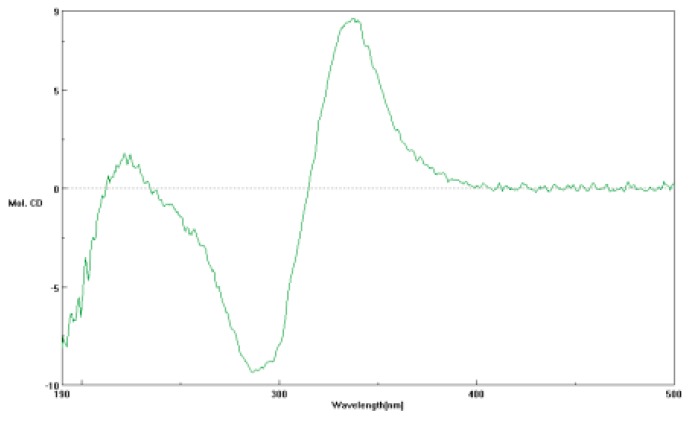
The CD spectrum of penicitroamide (**1**) in CH_3_OH.

The relative conformation of penicitroamide (**1**) was established by NOESY correlations. In the NOESY spectrum, the correlation from 3.35 (H-4) to 4.65 (H-5) proved the *cis* relationship between H-4 and H-5 (see Figure 2). Since compound **1** was a bridge-ring compound, the groups on the two bridgehead carbons must lie on the same side. The correlations from 4.65 (H-5) to 0.99 (H-28) and from 3.29 (H-9) to 1.07 (H-29) established the relative conformations of C-4, C-5, C-8 and C-9 (see Figure 2), meaning that the H-4, H-5, H-28, H-9 and H-29 were all a *cis* relationship in the core [5/6/5] ring systems. Unfortunately, the relative conformation of H-19 could not be determined using hints from the NOESY spectrum and the methyl group (H-30) located in the linear chain structure. The CD spectrum of penicitroamide (**1**) showed a positive Cotton effect at 360 (+7.3) and 220 (+1.4) nm and a negative Cotton effect at 281 (−7.8) nm (see Figure 3), and the value of specific optical rotation was −19 with the concentration of 0.01 mg/mL in the CH_3_OH solvent. Since the physical state is an oil, we cannot determine the absolute configuration of six chiral carbons using X-ray diffraction of penicitroamide (**1**), and only the relative configuration of penicitroamide (**1**) was reported in this paper.

### 2.2. Possible Biosynthesis of Penicitroamide *(**1**)*

Prolines play an important role in plants for responding to environmental and biotic stress [15]. When the plants underwent drought [16], saline [17], UV radiation [18], heavy metal pollution [19] and plant pathogen invasion [20,21], the amount of prolines massively accumulated. After proline accumulation, the endophytic fungi residing in the plants may take full advantage of the massive amount of prolines to synthesize secondary metabolites.

**Scheme 1:**
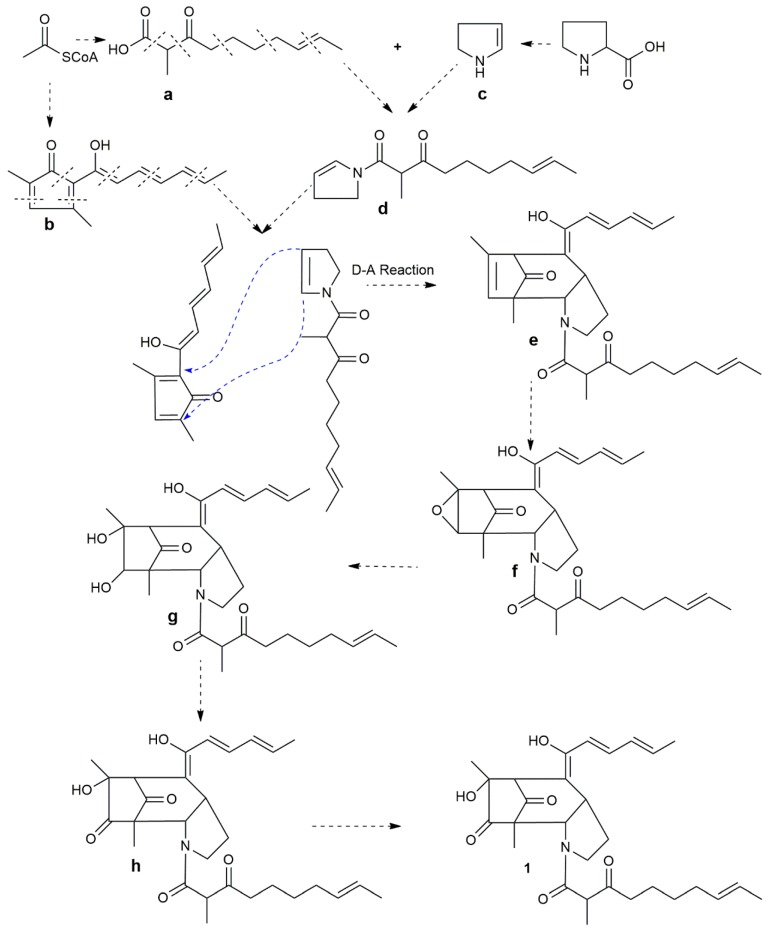
The possible biosynthesis pathway of penicitroamide (**1**).

Apparently, there was a proline core pyrrole ring in the structure of compound **1**; the pyrrole ring was possibly derived from proline by the decarboxylation reaction by the proline decarboxylase (see Scheme 1). The other parts of **1** can be decomposed into two polyketide parts, a and b (see Scheme 1). The two polyketide parts, a and b, could originate from the two six-acetyl coenzyme units in different binding modes, respectively. Furthermore, part a and part c could possibly be formed from the intermediate d by amidation. Then, the intermediates b and d could combine together to form the nucleus structure of **1** by the Diels-Alder reaction. Finally, the intermediate e could undergo epoxidation, epoxide cleavage, oxidation and hydroxylation resulting in compound **1**.

### 2.3. The Bioassay of Penicitroamide *(**1**)*

Compound **1** was evaluated against four cancer cell lines, A549, Caski, HepG2 and MCF-7, and the anticancer assay results showed no significant activity to the above four cancer cell lines with IC_50_ > 50 μg/mL. Then the antimicrobial assay against plant-pathogenic *Erwinia carotovora* subsp. Carotovora (Jones) Bersey et al. and *Sclerotium rolfsii* Sacc. were carried out using the well diffusion method; penicitroamide (**1**) displayed moderate inhibiting activity on two plant pathogens with MIC_50_ at 45 and 50 μg/mL. The positive control, mitomycin, was employed with MIC_50_ at 0.0.5–1 μg/mL. Finally, brine shrimp lethality assay was used in the laboratory, and the results showed 60% lethality to brine shrimp at 10 μg/mL.

## 3. Experimental Section

### 3.1. General Procedures

UV spectra were obtained on a SCINCO Spectrometer; IR spectra were recorded on Nicoler Auatar Spectrometer series FT360 spectrophotometer. 1D- and 2D-NMR spectra were recorded using a Bruker Ultrashield-400 MHz NMR spectrometer (Fällanden, Switzerland). Mass spectra were obtained on an EI mass spectrometer (Harrisburg, PA, USA). Silica gel GF254 (10–40 μm) was used for TLC and silica gel (200–300 mesh) for column chromatography (CC) were obtained from Qingdao Marine Chemical Factory (Qingdao, China). Fractions were monitored by TLC, and the spots were visualized under ultraviolet lamp with 254 and in iodine cylinder. Semi-preparative HPLC was performed on Dionex Ultra-3000 (Sunnyvale, CA, USA) and 1525 Waters (USA) using a Cosmosil C-18 column (10 μm × 20 mm × 250 mm and 5 μm × 4.6 mm × 250 mm). All organic solvents were analytical grade and were redistilled before use. The deuterium solvent DMSO-*d*_6_ was from CIL Company (Tewksbury, MA, USA). The CD spectrum was obtained on JASCO J-810 Spectrometer (Japan). The HPLC CH_3_OH and CH_3_CN were purchased from Tedia Company (Tianjin, China) and the water was purchased from WaHaHa (Wuhan, China) and YiBao Companies (Hangzhou, China).

### 3.2. Isolation and Identification of the Strain

The healthy leaves of *Tapiscia sinensis* Oliv. were collected in the Shennongjia National Forest Park and washed under running water, then immersed in the 5%–10% NaClO solution for 5 min, in 75% ethanol solution for 3 min, finally, washed by aseptic water three times, and then the aseptic water was removed by sterile filter papers. The sterilized leaves were cut into small pieces 0.5 cm × 0.5 cm^2^, and those pieces were placed on PDA plates in the incubator under 28 °C. After the microorganisms on the PDA plates were ready for purification, the growing fungi were repeatedly purified using the Streak method, until single colonies were obtained. The pure single colonies were then deposited on the slants under 4 °C. In total, 20 endophytic fungi were obtained from the leaves of *Tapiscia sinensis* Oliv., then those fungi were fermented in 200 mL PDB liquid medium with 500 mL Erlenmeyer flasks. The fungus (NO. 24) was selected for further chemical constituents investigation according to the results of the bioactivity assays and HPLC-DAD analysis-guiding. The fungus (NO. 24) was cultured in 50 L PDB liquid medium with 250,500 mL-Erlenmeyer flasks on the electronic oscillator under 28 °C with the speed at 120 r/min for 20 days. During the identification of the fungus NO. 24, we found that the 18S rDNA results shared 99% similarity to the fungus *Penicillium citrinum*, thus the specie of fungus NO. 24 was identified as *Penicillium citrinum*.

### 3.3. The Isolation and Purification of Penicitroamide *(**1**)*

The broth and mycelium were collected by eight layers of gauze, and the 50 L broth was extracted with 20 L ethyl acetate for five times, then the extract was condensed to a crude extract of 26 g under vacuum. Then 20 g of the crude extract was then subjected to silica gel column chromatography and eluted from light petroleum ether to ethyl acetate, then to methanol. The subfractions were merged into six fractions using TLC plates. The fraction D (0.5 g) was further subjected to silica gel column chromatography with the gradient elution from 30% petroleum ether/ethyl acetate to 40% petroleum ether/ethyl acetate in volume, then the fraction D was fractionated into two subfractions. And the subfraction D-1 (80 mg) was further purified by semi-preparative reverse-phase HPLC with constant elution using 45% acetronitrile-H_2_O to afford compound **1** (t_R_ 15.5 min, 4.5 mg).

Penicitroamide (**1**): brown oil, ${\left[\alpha \right]}_{D}^{25}$ = −19 (CH_3_OH, 0.02); UV (λ_max_): 225 (4.4), 285 (3.5) and 365 (2.8) nm; IR (KBr): 3430, 3012, 2966, 2930, 2880, 1820, 1800, 1721, 1686, 1624, 1520, 1460 cm^−1^; HR-EI-MS: 497.2775 (calcd. 497.2777); NMR data see Table 1. CD (*c* 0.01, CH_3_OH, 24 °C), 220 (+1.4), 281 (−7.8) and 360 (+7.3) nm.

### 3.4. Bioactivity Assays

#### 3.4.1. Cytotoxic Activity against Four Cancer Cell Lines In Vitro

A549, Caski, HepG2 and MCF-7 cells were cultured in RPMI 1640 medium (HyClone) supplemented with 10% FBS (HyClone). The cells were kept in 5% CO_2_ at 37 °C. The 3-(4,5-Dimethylthiazol-2-yl)-2, 5-diphenyl-2*H*-tetrazolium bromide (MTT, Sigma, Kawasaki, Japan) colorimetric assay was used to evaluate cell proliferation in the presence of different chemicals. The cells were seeded in 96-well culture plates and treated with desired concentrations of chemicals for a further 24 h. After treatment, the cells were incubated at 37 °C with MTT (10 μL/well, 5 mg/mL) for 4 h, and the cell growth response to the chemicals was determined by measuring the absorbance at 570 nm on a plate reader. Three replicates were used for each treatment. In the anticancer activity in vitro experiment, mitomycin was employed as positive control for the anticancer assay.

#### 3.4.2. Antimicrobial Assay

Antimicrobial assay against plant-pathogenic *Erwinia carotovora* subsp. Carotovora (Jones) Bersey et al. and *Sclerotium rolfsii* Sacc. were carried out using the well diffusion method [22]. Amphotericin B was used as positive control for the antimicrobial assay.

#### 3.4.3. Brine Shrimp Lethality Assay

The eggs of brine shrimp (*Artemia salina*) were purchased from Qingdao Haina Baichuan Biological Engineering Limited Company (Qingdao, China), and were incubated in artificial sea water. The artificial sea water contained 7.5 g bay salts in every liter of water, then was boiled and filtrated for the culture mediums of brine shrimp (*Artemia salina*). The 15 mg of eggs of brine shrimp were suspended in the 300 mL artificial sea water medium in a 500 mL flask for 48 h pumping in air and water, bathing at 25 °C. The hatchability of brine shrimp was between 85% and 88%. The 1 mg of penicitroamide (**1**) was dissolved in 200 μL DMSO. 10 μL, 5 μL and 2 μL of penicitroamide (**1**) was dropped into three wells containing 990 μL, 995 μL and 998 μL medium on a 24 wells plate, respectively. Each concentration sample was provided with three parallel samples. There was a DMSO control group and a medium blank group in a 24-well plate. There were 20 brine shrimps in every well. The 24-well plate containing the samples was incubated for 24 h at 25 °C. The lethality of brine shrimp and LC_50_ value were calculated according to Soli improved method.

## 4. Conclusions

Penicitroamide (**1**), a new metabolite with a new framework, was isolated from the endophytic fungus *Penicillium* sp. (NO. 24), isolated from the healthy leaves of *Tapiscia sinensis* Oliv. Penicitroamide (**1**) features a bicyclo[3.2.1]octane core unit with a high degree of carbonylization (four carbonyl groups and one enol group). The chemical structure of **1** was elucidated by analysis of 1D, 2D-NMR and MS data. In the bioassay, penicitroamide (**1**) displayed antibacterial potency to plant-pathogenic *Erwinia carotovora* subsp. Carotovora (Jones) Bersey et al. and *Sclerotium rolfsii* Sacc. with MIC_50_ at 45 and 50 μg/mL, and compound **1** also showed 60% lethality to brine shrimp at 10 μg/mL, but exhibited no significant activity toward A549, Caski, HepG2 and MCF-7 cells with IC_50_ > 50 μg/mL. Finally, the possible biosynthetic pathway of penicitroamide (**1**) was discussed; **1** possibly was evolved through the hybridization of prolines and the polyketide biosynthesis pathways by a key Diels-Alder reaction in *Penicillium* sp. (NO. 24).

## Figures and Tables

**Table 1:** The NMR data of compound **1** (100 MHz for ^13^C and 400 MHz for ^1^H in DMSO-*d*_6_) ^a^.

NO.	^13^C (DEPT135)	^1^H (*J* in Hz)	HMBC
2	47.6 (CH_2_)	3.71 (t, 9.5, 1H),2.75 (s, 1H)	5
3	29.2 (CH_2_)	2.10 (m, 1H), 1.50 (m, 1H)	5
4	34.9 (CH)	3.35 (m, 1H) ^d^	2, 5, 9, 23
5	61.2 (CH)	4.65 (d, 8.6, 1H)	2, 3, 9, 28
6	67.0 (C)	-	5, 28
7	208.5 (C)	-	5, 9, 8-OH, 28, 29
8	73.2 (C)	-	8-OH, 9, 29
9	45.3 (CH)	3.29 (m, 1H) ^d^	3, 5, 8-OH, 29
10	108.9 (C)	-	13, 4, 9
11	197.3 (C)	-	5, 9, 28
12	167.8 (C)	-	13, 14
13	118.6 (CH)	6.66 (d, 14.6, 1H)	15
14	142.3 (CH)	7.27 (dd, 11.0, 14.0, 1H)	13, 15, 16
15	131.1 (CH) ^b^	6.39 (d, 11.4, 1H)	13, 16, 17
16	139.7 (CH)	6.28 (m, 1H)	14, 15, 17
17	18.6 (CH_3_)	1.85 (d, 6.0, 3H)	15, 16
18	170.7 (C)	-	2, 5, 19, 30
19	50.9 (CH)	3.83 (br d, 6.8, 1H)	30
20	206.8 (C)	-	19, 21, 22, 30
21	40.0 (CH_2_)	2.39 (m, 2H)	22, 23
22	22.5 (CH_2_)	1.39 (m, 2H)	21, 23, 24
23	28.3 (CH_2_)	1.23 (m, 2H)	21, 22, 24, 27
24	31.8 (CH_2_)	1.89 (m, 2H)	22, 23, 25
25	131.1 (CH)	5.36 (s, 1H)	23, 24, 27
26	124.5 (CH)	5.36 (s, 1H) ^c^	24, 27
27	17.7 (CH_3_)	1.59 (br s, 3H) ^c^	25
28	10.4 (CH_3_)	0.99 (s, 3H)	5
29	23.4 (CH_3_)	1.07 (s, 3H)	8-OH, 9
30	13.3 (CH_3_)	1.11 (d, 6.1, 3H)	19

^a^ The 8-OH and 12-OH were observed at δ 6.12 and 14.1, respectively; ^b^ The chemical shifts of C-15 and C-25 were overlapped in ^13^C-NMR spectrum; ^c^ The chemical shifts of H-25 and H-26 were overlapped in ^1^H-NMR spectrum; ^d^ The peaks of H-4 and H-9 were partly overlapped with water peak in ^1^H-NMR spectrum.

## Supplementary Materials

Supplementary materials can be accessed at: http://www.mdpi.com/1420-3049/21/11/1438/s1.
