# Supplementary material for: Penicitroamide, an Antimicrobial Metabolite with High Carbonylization from the Endophytic Fungus Penicillium sp. (NO. 24)
Source: Molecules. 2016 Oct 28;21(11):1438. doi: 10.3390/molecules21111438 (PMC6274507; doi:10.3390/molecules21111438)
Supplement: Supplementary file 1 [file molecules-21-01438-s001.pdf]

Supplementary Materials: Penicitroamide, an Antimicrobial Metabolite with High Carbonylization from the Endophytic Fungus *Penicillium* sp. (NO. 24)

Zi-Wei Feng, Meng-Meng Lv, Xue-Shuang Li, Liang Zhang, Cheng-Xiong Liu, Zhi-Yong Guo, Zhang-Shuang Deng, Kun Zou and Peter Proksch

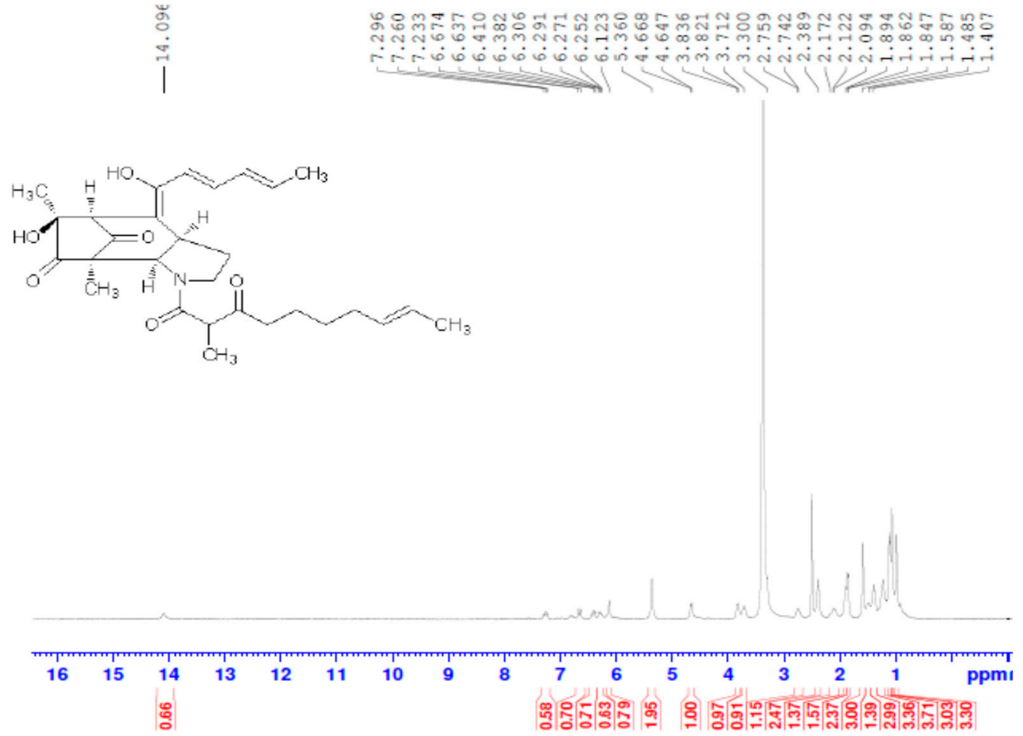

Figure S1. The ^{1}H NMR spectrum of Penicitroamide (**1**).

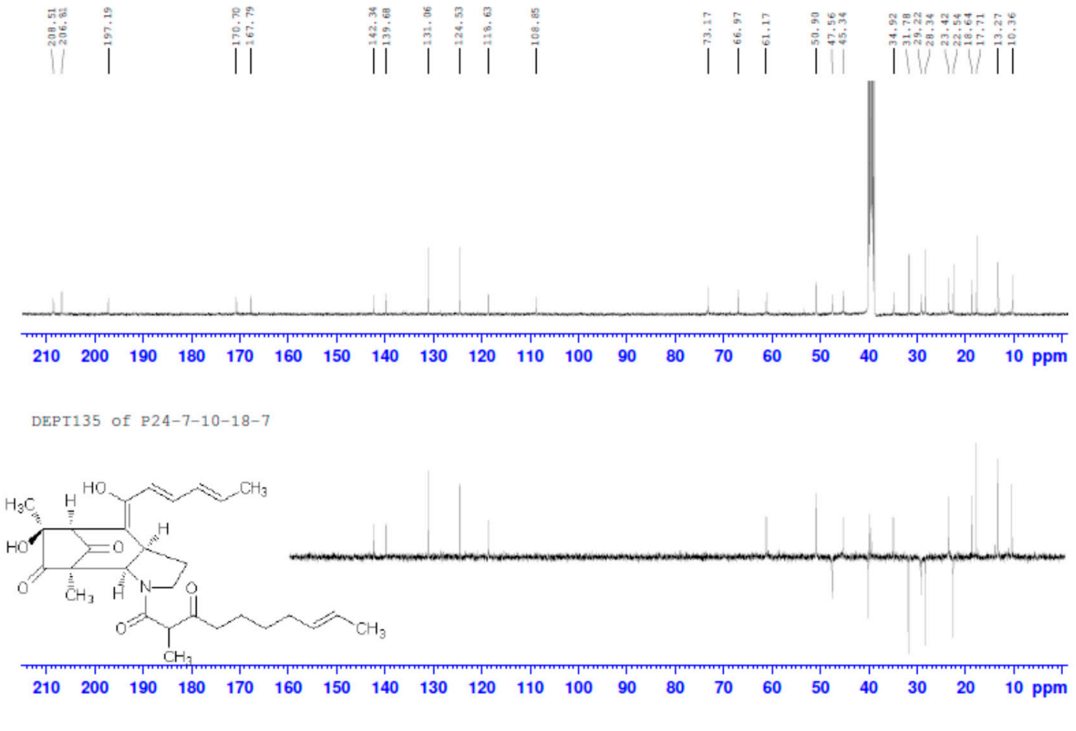

Figure S2. The ^{13}C and DEPT135 NMR spectra of Penicitroamide (**1**).

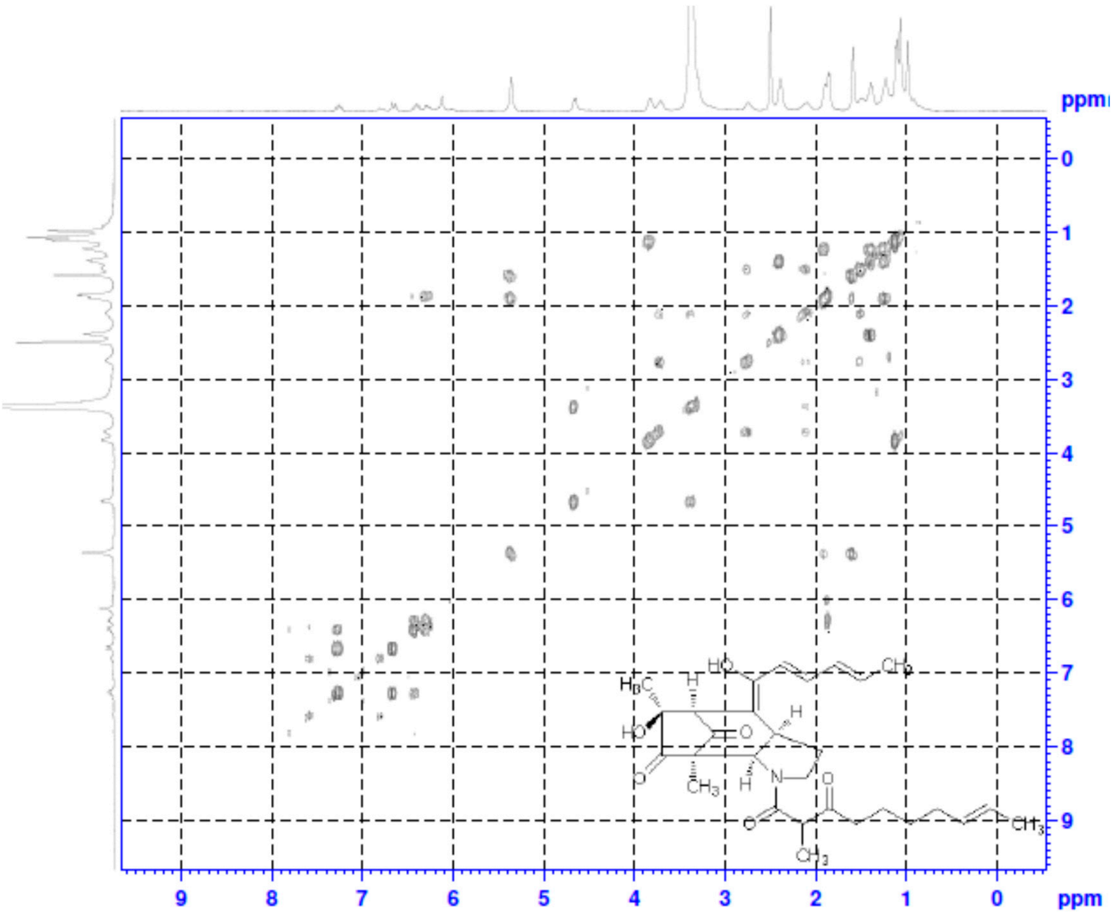

Figure S3. The COSY NMR spectrum of Penicitroamide (**1**).

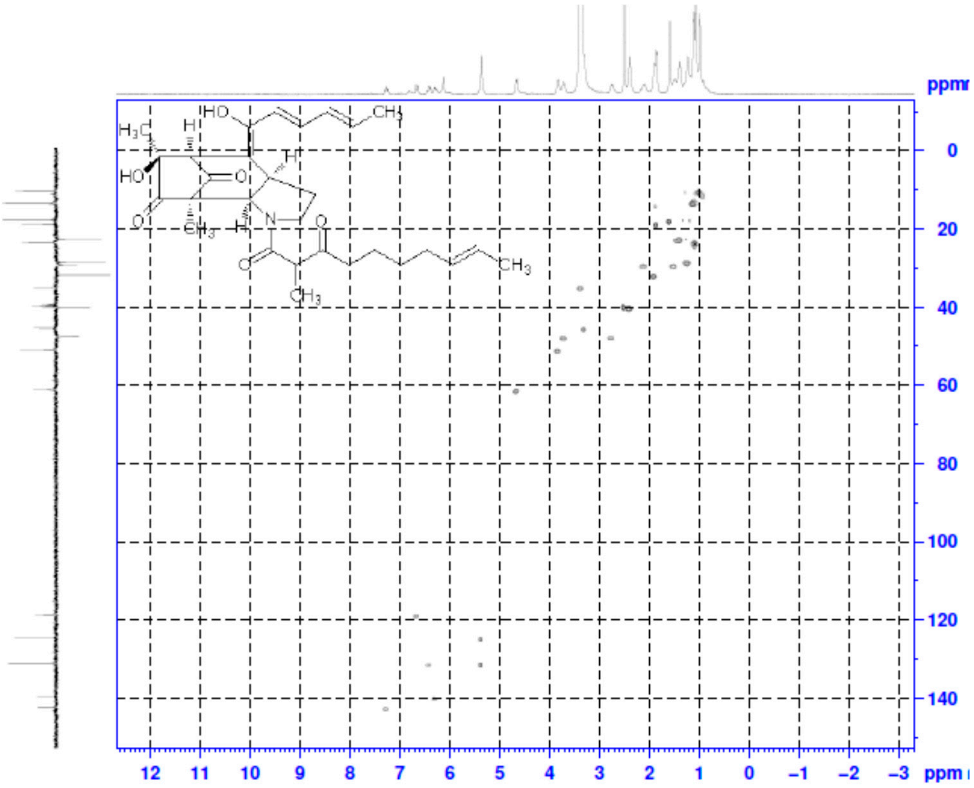

Figure S4. The HSQC NMR spectrum of Penicitroamide (**1**).

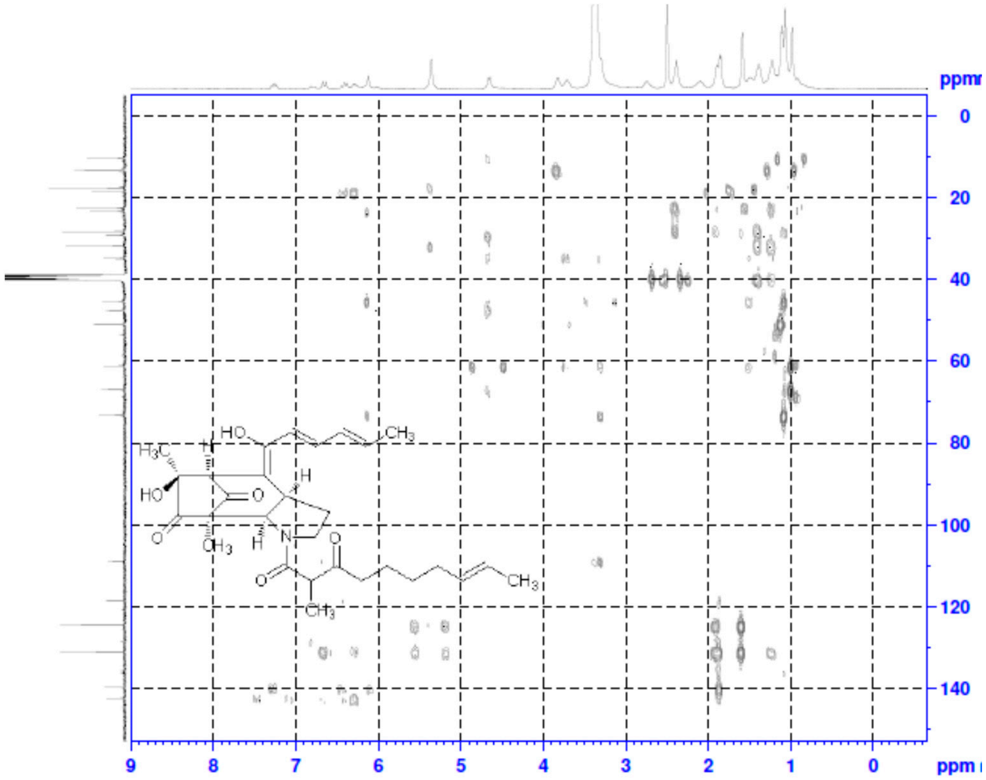

Figure S5. The HMBC NMR spectrum of Penicitroamide (**1**).

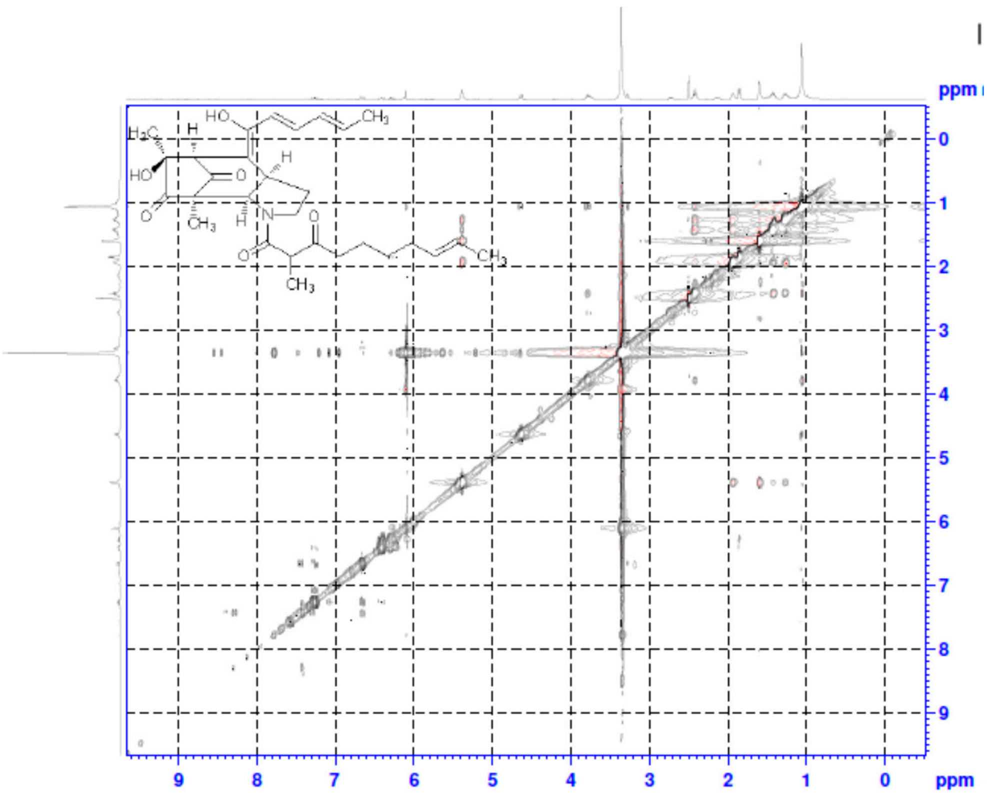

Figure S6. The NOESY NMR spectrum of Penicitroamide (**1**).

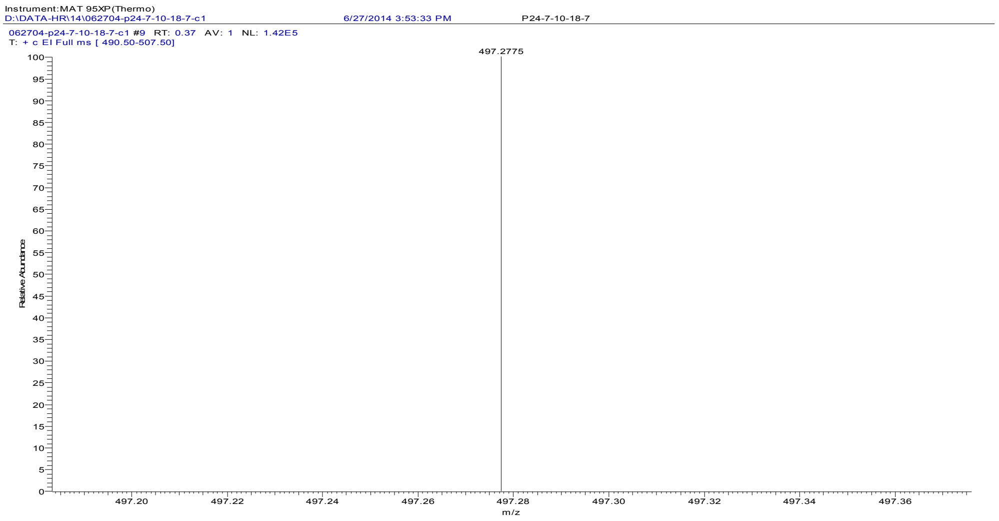

Figure S7. The HR-EI-MS of Penicitroamide (**1**).

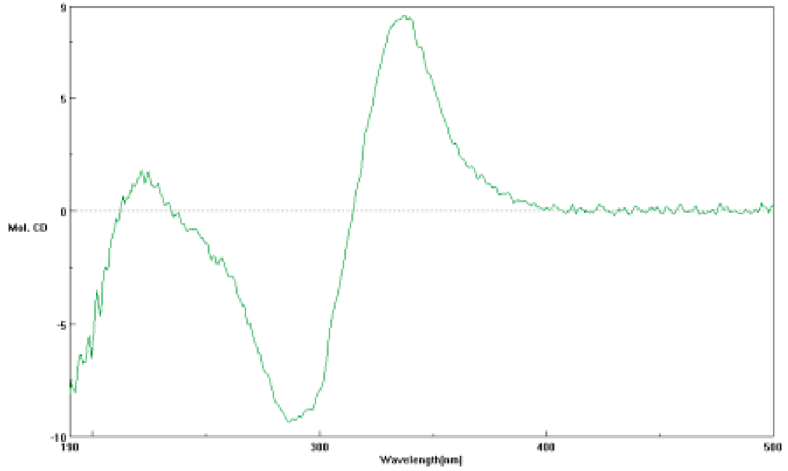

Figure S8. The CD spectrum of Penicitroamide (**1**).
